# Occupation and COVID-19 diagnosis, hospitalisation and ICU admission among foreign-born and Swedish-born employees: a register-based study

**DOI:** 10.1136/jech-2021-218278

**Published:** 2022-01-07

**Authors:** Chioma Adanma Nwaru, Ailiana Santosa, Stefan Franzén, Fredrik Nyberg

**Affiliations:** 1 School of Public Health and Community Medicine, Institute of Medicine, Sahlgrenska Academy, University of Gothenburg, Gothenburg, Sweden; 2 National Diabetes Register, Centre of Registers Västra Götaland, Gothenburg, Sweden

**Keywords:** COVID-19, epidemiology, public health

## Abstract

**Background:**

Research on occupation and risk of COVID-19 among foreign-born workers is lacking. We investigated whether working in essential occupations was associated with COVID-19 diagnosis, hospitalisation and intensive care unit (ICU) admission and whether foreign-born workers in similar occupations as Swedish-born individuals had a higher risk of the studied outcomes.

**Methods:**

Occupational data (2018–2019) of 326 052 employees (20–65 years) who were resident in Sweden as of 1 January 2020 were linked to COVID-19 data registered from 1 January 2020 to 28 February 2021. We analysed the risk of COVID-19 outcomes in different occupational groups and in four immigrant/occupation intersectional groups using Cox proportional hazards regression with adjustments for sociodemographic and socioeconomic characteristics and pre-existing comorbidities.

**Results:**

We identified 29797, 1069 and 152 cases of COVID-19 diagnosis, hospitalisations and ICU admissions, respectively, in our cohort. Workers in essential occupations had an elevated risk of COVID-19 diagnosis, hospitalisation, and ICU admissions. Healthcare workers had a higher risk of all the outcomes compared with other essential workers. Relative to Swedish-born workers in non-essential occupations, foreign-born workers in essential occupations had 1.85 (95% CI 1.78 to 1.93), 3.80 (95% CI 3.17 to 4.55) and 3.79 (95% CI 2.33 to 6.14) times higher risk of COVID-19 diagnosis, hospitalisation and ICU admission, respectively. The corresponding risks among Swedish-born workers in essential occupations were 1.44 (95% CI 1.40 to 1.49), 1.30 (95% CI 1.08 to 1.56) and 1.46 (95% CI 0.90 to 2.38).

**Conclusion:**

Occupation was associated with COVID-19 outcomes and contributed to the burden of COVID-19 among foreign-born individuals in this study.

## Introduction

Sweden has had higher rates of COVID-19 infection and deaths than other Nordic countries.^1^ As of 28 November 2021, more than a million COVID-19 cases have been reported in Sweden, with over 15 000 deaths.^2^ Analyses of the risk of different COVID-19 outcomes within the Swedish population shows unequal distribution, with a higher risk in foreign-born individuals than in Swedish-born population.^3 4^ Studies from Norway,^5^ Spain^6^ and the USA^7^ also report increased risk of COVID-19 among immigrants and minority groups. However, the mechanisms underlying the disproportionate risk among foreign-born individuals remain incompletely understood.

Occupational interactions is a well-known determinant of infectious disease transmission,^8–10^ which explains why during the COVID-19 pandemic, several countries encouraged remote working as one of the strategies for containing the spread of SARS-CoV-2, the virus that causes COVID-19. However, not all types of work can be performed remotely. Work in many so-called essential occupations, that is, in industries and organisations that are critical for the functioning of societal infrastructures, demand on-site labour and involves close proximity with members of the public and coworkers.^11^ Essential occupations typically include work in the healthcare sector, service sector, transport services, security services and cleaning services. Previous research has shown that workers in essential occupations have a higher risk of COVID-19 than those in non-essential occupations and among the essential workers, those in the healthcare sector are reported to have the greatest risk.^12–16^

In many Western countries, foreign-born individuals are overrepresented in less well paid essential occupations that are often characterised by poor working conditions and elevated risk of infection.^17–19^ Analysing occupational risk of COVID-19 among the foreign-born population could shed further light on both the determinants of the infection and the right groups to target in working to reduce COVID-19 cases and associated deaths. However, the contribution of occupation to the risk of COVID-19 among foreign-born individuals in Sweden has not been studied. Two studies that have investigated this topic come from the US^20^ and Norway^21^ and provide conflicting evidence. The studies are also limited in focusing on specific groups of immigrants, considering only COVID-19 infection onset, and accounting for a limited number of potential confounders.

Therefore, using nationwide register data, we examine whether working in essential occupations is associated with COVID-19 diagnosis, hospitalisation and intensive care unit (ICU) admission and whether foreign-born workers in similar occupations as Swedish-born individuals have a higher risk of the outcomes.

## Methods

### Study design and population

**Figure 1:**
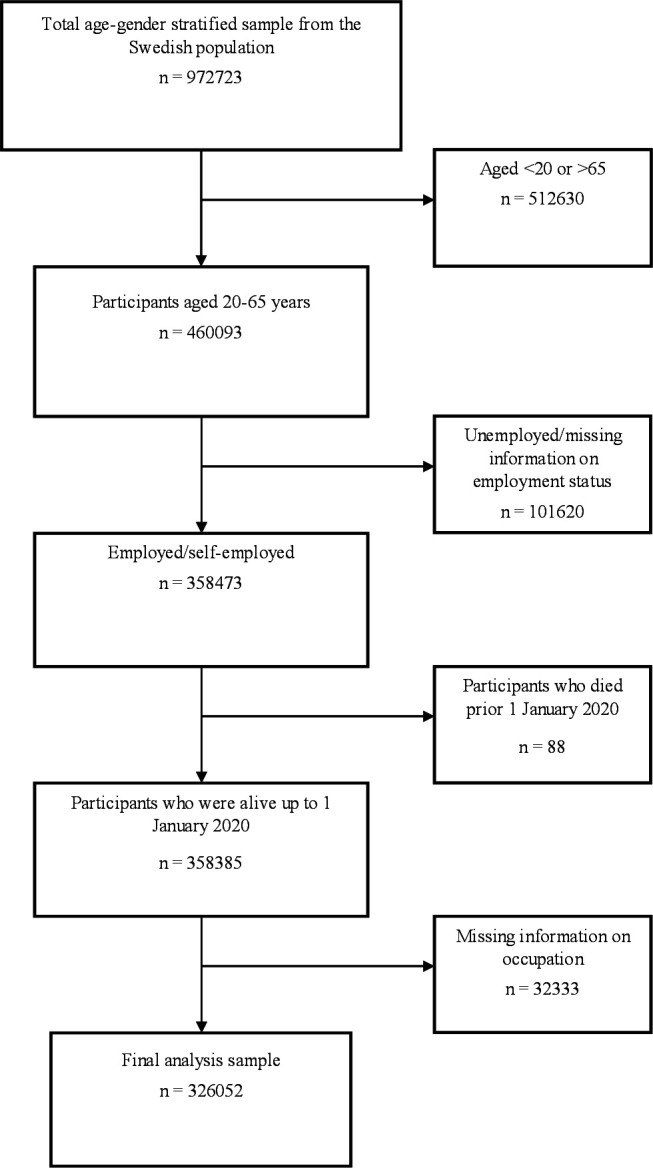
Flow chart illustrating selection of the study population of individuals aged 20–65 years who were employed or self-employed.

This study is part of the SCIFI-PEARL (Swedish COVID-19 Investigation for Future Insights-a Population Epidemiology Approach using Register Linkage) project, a nationwide multiregister-based observational study designed in response to the COVID-19 pandemic. Details about the project have been published elsewhere.^22^ Briefly, the project includes regularly updated data of all individuals with COVID-19 identified from different registers in Sweden. This includes individuals with positive SARS-CoV-2 polymerase chain reaction (PCR) test results identified from the national database of notifiable diseases (SmiNet), individuals identified from the National Patient Register (NPR) or Cause-of-Death Register, individuals identified from the Intensive Care Register, and individuals identified from primary care data from the Stockholm Region and Region Västra Götaland. The project also includes data for a comparison cohort (N=972 723) created by means of a stratified random sample of all individuals resident in Sweden on 1 January 2020. For this study, we selected, from the comparison cohort, all individuals who were aged 20–65 years and were employed or self-employed (n=358 385) based on employment information (2018–2019) obtained from the Longitudinal Integrated Database for Health Insurance and Labour Market Studies (LISA). All individuals with missing information on occupation were excluded (n=32 333), leaving a final analysis sample of 326 052 individuals (figure 1).

### Measurements

Data on sociodemographic and socioeconomic characteristics obtained from the LISA register and on pre-existing comorbidities retrieved from the NPR were linked to the study population using a personal identification number and pseudonymised.

#### Exposure variables

Information on country of birth was used to discriminate between foreign-born and Swedish-born individuals. Swedish-born individuals comprised all study subjects who were born in Sweden, including those who were born to immigrant parents residing in Sweden. Occupation was registered as a four-digit occupational code according to the Swedish Standard Occupational Classification (SSYK2012) and used to group the study subjects into essential and non-essential occupations based on similar criteria as found in Billingsley *et al* 23 (online supplemental table A1). Essential workers included healthcare workers, teachers, service sector workers (ie, sales workers, food processing and related trade workers, and food preparation assistants), police and security services, postal and delivery workers, cleaners, and taxi, bus, and tram drivers. The last four categories were classified as a group and defined as ‘other essential workers’ in the analysis to enhance statistical power. All those who did not belong to any of the selected essential occupations were defined as non-essential workers and used as the reference category in the analysis for the first research question. For the second research question, we constructed a four-category intersection variable using the information on immigrant status and occupation. The categories were Swedish-born workers in non-essential occupations (n=197 565, 61%) (reference category), Swedish-born workers in essential occupations (n=69 283, 21%), foreign-born workers in non-essential occupations (n=36 392, 11%) and foreign-born workers in essential occupations (n=22 812, 7%).

10.1136/jech-2021-218278.supp1 Supplementary data

#### Outcome variables and follow-up

We investigated three outcomes:

1. COVID-19 diagnosis, referring to all individuals in the study population who had specialist healthcare encounter (visit or hospitalisation) with a code of COVID-19 (International Classification of Diseases, version 10 (ICD-10)-SE U07.1 and U07.2) in the NPR or the same codes as underlying or contributing cause of death in the cause-of-death register, or a positive test result for SARS-CoV-2 in SmiNet. The event date was the earliest of these.
2. COVID-19 hospitalisation, referring to all individuals admitted to the hospital based on primary or secondary diagnosis for COVID-19. The event date was the date of hospital admission.
3. ICU admission, referring to all those who were transferred to or were directly treated in the ICU based on data from the Swedish Intensive Care register. The event date was the date of ICU admission.

For each outcome, we followed the participants starting from 1 January 2020 to the earliest of outcome, emigration, death or end of follow-up, which was 28 February 2021.

#### Potential confounders

Selection of potential confounders was informed by literature^3 4^ and included age (20–34, 35–44, 45–54 and 55–65), gender (men, women), healthcare region (Stockholm, Northern, Southeastern, Southern, Uppsala-Orebro and Western), marital status (married/cohabiting, single and separated/divorced/widowed), highest education (primary, secondary and tertiary), individual annual gross income (<SEK1000, SEK1000–SEK2999, SEK3000–SEK4999 and ≥SEK5000) and pre-existing comorbidities, such as hypertension, diabetes, stroke, obesity, asthma, chronic obstructive pulmonary disease, pneumonia and psychiatric conditions. The ICD-10 codes of these comorbidities are provided as online supplemental table A2.

10.1136/jech-2021-218278.supp2 Supplementary data

### Statistical analysis

We summarised sample characteristics using frequencies and percentages, and used the χ² test for differences between groups. Incidence rates (cases per 1000 person-years) of COVID-19 diagnosis, hospitalisation and ICU admission, were calculated by immigrant status and by occupational groups. Cox proportional hazards regression was used to assess the risk of the outcomes in different occupational groups and across the immigrant/occupation intersectional groups. We ran four separate models: an unadjusted model and three adjusted models. In the first adjusted model (model I), we controlled for age, gender, marital status, immigrant status (if relevant), and healthcare region (dichotomised into Stockholm and other regions). We added education and income in Model II, and pre-existing comorbidities in the final model III. All covariates were treated as categorical variables when we estimated the risk of COVID-19 diagnosis and hospitalisation. For the risk of COVID-19-related ICU admission, due to few ICU events, age and income were treated as continuous variables. We expressed the coefficients as HR with 95% CI, and statistical significance was defined as a two-sided p<0.05.

To assess the magnitude and statistical significance of the interaction between occupation and immigrant status, we estimated three measures of additive interactions: the relative excess risk due to interaction (RERI), the attributable proportion due to interaction (AP) and the synergy index (SI). CIs and p values were calculated using the delta method,^24^ and RERI >0, AP >0 or SI >1 were interpreted as indicating additive interaction, that is, that the combined effects of the two exposure variables is larger (or smaller) than the sum of the individual effects of the two exposures.^25^ All statistical analyses were performed with STATA V.16.

## Results

We studied 326 052 individuals with a mean (SD) age of 43 (12.53) years. Table 1 presents the characteristics of the sample and the differences in characteristics between foreign-born and Swedish-born population groups. Half of the sample were women, 18% were of foreign-born background, and 28% worked in essential occupations. Foreign-born individuals were more often in essential occupations than Swedish-born individuals (38% vs 26%). Other differences between the groups are shown in table 1. Until 28 February 2021, we identified 29 797 cases of COVID-19 diagnosis, 1069 cases of COVID-19-related hospitalisations, and 152 cases of COVID-19-related ICU admission in the total sample. The incidence rate per 1000 person-years among the foreign-born group was 100.0 for COVID-19 diagnosis, 6.3 for hospitalisation and 0.8 for ICU admission. Among the Swedish-born population, the rates were lower, at 75.8, 2.0 and 0.3 for diagnosis, hospitalisation and ICU admission, respectively.

**Table 1:** Distribution of sample characteristics and differences in characteristics between foreign-born and Swedish-born aged 20–65 years and employed or self-employed

	Total sample	Swedish-born	Foreign-born	P value
	N=326 052	N=266 848	N=59 204	
	n (%)	%	%	
Age (years)				<0.001
20–34	92 361 (28.33)	28.62	27.01	
35–44	75 903 (23.28)	21.72	30.31	
45–54	79 274 (24.31)	24.57	23.14	
55–65	78 514 (24.08)	25.09	19.54	
Gender				<0.001
Men	162 143 (49.73)	49.55	50.52	
Women	163 909 (50.27)	50.45	49.48	
Healthcare region				<0.001
Stockholm	79 217 (24.30)	22.12	34.08	
Northern	28 793 (8.83)	9.74	4.73	
Southeastern	34 187 (10.49)	10.88	8.71	
Southern	56 123 (17.21)	17.03	18.03	
Uppsala-Orebro	65 416 (20.06)	20.95	16.07	
Western	62 316 (19.11)	19.28	18.37	
Marital status				<0.001
Married	140 663 (43.14)	41.05	52.58	
Single	146 891 (45.05)	48.36	30.15	
Divorced/widowed	38 498 (11.81)	10.59	17.27	
Highest education				<0.001
Primary	26 574 (8.15)	6.95	14.21	
Secondary	153 214 (46.99)	49.12	39.28	
Tertiary	143 512 (44.02)	43.94	46.51	
Missing	2752 (0.84)			
Individual annual gross income				<0.001
<SEK1000	21 334 (6.54)	6.23	7.94	
SEK1000–SEK2999	92 493 (28.37)	26.37	37.37	
SEK3000–SEK4999	152 067 (46.64)	47.68	42.97	
≥SEK5000	60 158 (18.45)	19.72	12.72	
Broad occupational groups				<0.001
Non-essential workers	233 957 (71.75)	74.04	61.47	
Essential workers	92 095 (28.25)	25.96	38.53	
Specific occupational groups				<0.001
Non-essential workers	233 957 (71.75)	74.04	61.47	
Healthcare workers	29 233 (8.97)	8.01	13.26	
Teachers	26 642 (8.17)	8.16	8.21	
Service sector workers	22 957 (7.04)	6.88	7.78	
Police and security services	3230 (0.99)	1.06	0.66	
Postal workers and delivery	1818 (0.56)	0.56	0.53	
Taxi, bus, and tram drivers	2778 (0.85)	0.49	2.47	
Cleaners	5437 (1.67)	0.79	5.61	
Hypertension				<0.001
No	314 789 (96.55)	96.46	96.94	
Yes	11 263 (3.45)	3.54	3.06	
Diabetes				<0.001
No	314 295 (96.39)	96.61	95.44	
Yes	11 757 (3.61)	3.39	4.56	
Obesity				0.604
No	320 330 (98.25)	98.25	98.22	
Yes	5722 (1.75)	1.75	1.78	
Stroke				<0.001
No	325 005 (99.68)	99.66	99.76	
Yes	1047 (0.32)	0.34	0.24	
Pneumonia				0.224
No	322 709 (98.97)	98.98	98.93	
Yes	3343 (1.03)	1.02	1.07	
COPD				0.749
No	325 446 (99.81)	99,81	99.82	
Yes	606 (0.19)	0.19	0.18	
Asthma				<0.001
No	321 156 (98.50)	98.42	98.83	
Yes	4896 (1.50)	1.58	1.17	
Psychiatric conditions				<0.001
No	316 291 (97.01)	96.91	97.42	
Yes	9761 (2.99)	3.09	2.58	

COPD, chronic obstructive pulmonary disease.

Table 2 illustrates the association between occupation and COVID-19 diagnosis, hospitalisation and ICU admission. Working in essential occupation was associated with an increased risk of COVID-19 diagnosis and hospitalisation already in the unadjusted model and adjustment for potential confounding factors only marginally affected the estimates. When comparing the risk of COVID-19 diagnosis in different groups of essential workers, healthcare workers had the highest risk, followed by teachers, service sector workers and ‘other essential workers’. In contrast, for the more severe outcomes of COVID-19 hospitalisation and ICU admission, healthcare workers and ‘other essential workers’ were the groups showing elevated risk, while teachers and service sector workers did not have any statistically significant increased risk.

**Table 2:** Association between occupation and COVID-19 outcomes among individuals aged 20–65 years and employed or self-employed

	Unadjusted model	Model I†	Model II‡	Model III§
	HR (95% CI)	HR (95% CI)	HR (95% CI)	HR (95% CI)
COVID-19 diagnosis				
Broad occupational groups				
Non-essential workers	1	1	1	1
Essential workers	1.50 (1.46 to 1.53)	1.42 (1.39 to 1.46)	1.44 (1.41 to 1.48)	1.44 (1.41 to 1.48)
Specific occupational groups				
Non-essential workers	1	1	1	1
Healthcare workers	2.00 (1.94 to 2.07)	1.93 (1.87 to 2.00)	1.92 (1.86 to 1.99)	1.92 (1.85 to 1.99)
Teachers	1.46 (1.40 to 1.51)	1.41 (1.36 to 1.47)	1.44 (1.38 to 1.50)	1.44 (1.38 to 1.50)
Service sector workers	1.13 (1.08 to 1.19)	1.09 (1.04 to 1.14)	1.10 (1.05 to 1.15)	1.10 (1.05 to 1.15)
Other essential workers*	1.13 (1.07 to 1.20)	1.07 (1.01 to 1.13)	1.09 (1.02 to 1.15)	1.08 (1.02 to 1.15)
COVID-19 hospitalisation				
Broad occupational groups				
Non-essential workers	1	1	1	1
Essential workers	1.35 (1.19 to 1.53)	1.43 (1.25 to 1.63)	1.36 (1.19 to 1.57)	1.36 (1.19 to 1.56)
Specific occupational groups				
Non-essential workers	1	1	1	1
Healthcare workers	1.64 (1.37 to 1.96)	1.74 (1.44 to 2.11)	1.74 (1.43 to 2.11)	1.70 (1.41 to 2.06)
Teachers	1.00 (0.80 to 1.27)	1.20 (0.95 to 1.52)	1.18 (0.93 to 1.50)	1.19 (0.94 to 1.52)
Service sector workers	0.85 (0.65 to 1.11)	1.07 (0.82 to 1.41)	0.97 (0.73 to 1.28)	0.98 (0.74 to 1.30)
Other essential workers*	2.28 (1.83 to 2.84)	1.60 (1.28 to 2.00)	1.46 (1.16 to 1.84)	1.45 (1.15 to 1.82)
COVID-19 ICU admissions				
Broad occupational groups				
Non-essential workers	1	1	1	1
Essential workers	1.24 (0.89 to 1.75)	1.62 (1.33 to 2.32)	1.49 (1.03 to 2.14)	1.47 (1.02 to 2.11)
Specific occupational groups				
Non-essential workers	1	1	1	1
Healthcare workers	1.33 (0.80 to 2.23)	1.82 (1.06 to 3.15)	1.86 (1.07 to 3.23)	1.80 (1.04 to 3.12)
Teachers	0.69 (0.33 to 1.41)	1.07 (0.51 to 2.23)	1.06 (0.50 to 2.23)	1.06 (0.50 to 2.24)
Service sector workers	0.60 (0.26 to 1.36)	1.00 (0.44 to 2.30)	0.82 (0.36 to 1.90)	0.82 (0.36 to 1.91)
Other essential workers*	3.29 (2.01 to 5.37)	2.34 (1.41 to 3.88)	1.98 (1.19 to 3.30)	1.94 (1.16 to 3.23)

HR and 95% CI obtained from COX proportional hazards regression.

*Other essential workers comprised police and security services, postal workers and delivery, taxi, bus and tram drivers and cleaners.

†Adjusted for age, gender, marital status, immigrant status and healthcare region.

‡Model I+education and income.

§Model II+pre-existing comorbidities (hypertension, diabetes, obesity, stroke, asthma, COPD, pneumonia and psychiatric conditions).

COPD, chronic obstructive pulmonary disease.

Compared with the Swedish-born population, foreign-born individuals had a higher risk of all three COVID-19 outcomes following adjustment for potential confounding factors, including occupation (online supplemental table A3). Table 3 shows the association between immigrant/occupation intersection and all three outcomes, with Swedish-born workers in non-essential occupations as the reference group. For simplicity, we present only the result of the fully adjusted model. Regarding COVID-19 diagnosis, foreign-born workers in essential occupations had the highest risk, followed by Swedish-born workers in essential occupations, and then foreign-born workers in non-essential occupations. The result for hospitalisation showed higher HRs among foreign-born workers in essential (HR 3.80, 95% CI 3.17 to 4.55) and non-essential (HR 2.64, 95% CI 2.24 to 3.11) occupations relative to Swedish-born workers in essential occupations (HR 1.30, 95% CI 1.08 to 1.56). The same pattern was observed for ICU admission, with foreign-born workers in essential occupations remaining the group with the highest risk.

10.1136/jech-2021-218278.supp3 Supplementary data

**Table 3:** Association between immigrant/occupation intersection and COVID-19 outcomes among individuals aged 20–65 years and employed or self-employed

	Occupational groups		HR (95% CI) for workers in essential occupations within strata of immigrant status	P value for multiplicative interaction
	Non-essential occupations	Essential occupations		
	HR (95% CI)	HR (95% CI)		
COVID-19 diagnosis				
Swedish-born	1	1.44 (1.40 to 1.49) P≤0.001	1.45 (1.41 to 1.50) P≤0.001	0.973
Foreign-born	1.28 (1.23 to 1.33) P≤0.001	1.85 (1.78 to 1.93) P≤0.001	1.42 (1.35 to 1.50) P≤0.001	
HR (95% CI) for foreign-born within strata of occupation	1.26 (1.21 to 1.31) P≤0.001	1.32 (1.26 to 1.38) P≤0.001		
Measures of interaction on addictive scale:				
RERI (95% CI), 0.12 (0.04 to 0.21); p=0.005				
AP (95% CI), 0.07 (0.02 to 0.11); p=0.003				
SI (95% CI), 1.17 (1.05 to 1.31); p=0.005				
COVID-19 hospitalisation				
Swedish-born	1	1.30 (1.08 to 1.56) P=0.006	1.24 (1.02 to 1.50) P=0.026	0.445
Foreign-born	2.64 (2.24 to 3.11) P≤0.001	3.80 (3.17 to 4.55) P≤0.001	1.47 (1.21 to 1.80) P≤0.001	
HR (95% CI) for foreign-born within strata of occupation	2.54 (2.15 to 3.00) P≤0.001	3.06 (2.44 to 3.82) P≤0.001		
Measures of interaction on addictive scale:				
RERI (95% CI), 0.86 (0.19 to 1.53); p=0.011				
AP (95% CI), 0.23 (0.07 to 0.38); p=0.003				
SI (95% CI), 1.45 (1.09 to 1.92); p=0.011				
COVID-19 ICU admissions*				
Swedish-born	1	1.46 (0.90 to 2.38) P=0.122		0.940
Foreign-born	2.56 (1.66 to 3.95) P≤0.001	3.79 (2.33 to 6.14) P≤0.001		
Measures of interaction on addictive scale:				
RERI (95% CI), 0.76 (–1.07 to 2.59); p=416				
AP (95% CI), 0.20 (–0.22 to 0.62); p=0.353				
SI (95% CI), 1.37 (0.64 to 2.93); p=0.411				

HR and 95% CI obtained from COX proportional hazards regression.

HR adjusted for age, gender, marital status, healthcare region, education, income and pre-existing comorbidities (hypertension, diabetes, obesity, stroke, asthma, COPD, pneumonia and psychiatric condition).

*HR (95%CI) for foreign-born within strata of occupation and HR (95% CI) for workers in essential occupations within strata of immigrant status could not be estimated due to few ICU events in each stratum.

AP, attributable proportion due to interaction; COPD, chronic obstructive pulmonary disease; ICU, intensive care unit; RERI, relative excess risk due to interaction; SI, Synergy Index.

The intersectional model indicated no significant departure from multiplicativity. Accordingly, measures of additive interaction indicated positive additive interaction between occupation and immigrant status in association with COVID-19 diagnosis, and more strongly with hospitalisation (table 3), with very similar interaction as for hospitalisation suggested for the ICU outcome, although not statistically significant due to the small sample size with reduced power. For example, the AP due to interaction for hospitalisation was estimated to be 0.23, suggesting that 23% of COVID-19-related hospitalisation in foreign-born workers in essential occupations was due to the interaction itself, that is, beyond what would be expected from independent risks.

## Discussion

In this study, working in essential occupations was associated with an elevated risk of COVID-19 diagnosis, hospitalisation and ICU admission, and the risk was markedly higher for workers in the healthcare sector compared with individuals in other essential occupations. When we compared the risk of COVID-19 diagnosis, hospitalisation and ICU admission across the immigrant/occupation intersectional groups, we found that, of all the groups, foreign-born workers in essential occupations had the highest risk for all three outcomes, while Swedish-born workers in non-essential occupations had the lowest risk. Foreign-born workers in non-essential occupations had lower HR for COVID-19 diagnosis and higher HRs for COVID-19 hospitalisation and ICU admission relative to Swedish-born workers in essential occupations. The result of the interaction tests indicated that the joint effect of occupation and immigrant status in association with COVID-19 diagnosis and hospitalisation was larger than the sum of the estimated effect of occupation alone and immigrant status alone, that is, that some proportion of health effects was beyond what would be expected from independent risks.

Our finding of increased risk of COVID-19 outcomes among employees in essential occupations corroborates previous studies from other countries,^12–14 20^ and likewise the finding of higher risk among healthcare workers as compared with other essential workers.^12 14^ Zhang^12^ showed that the risks of disease exposure and physical proximity to persons with COVID-19 are greater in healthcare workers than in other essential workers, which could explain their higher risk of COVID-19 outcomes. Here, we found that teachers had the second highest risk of COVID-19 diagnosis, which is in disagreement with finding reported by the Swedish Public Health Agency.^26^ This contrasting finding might be because the study from the Swedish Public Health Agency was restricted to COVID-19 cases identified as test positive only from the SmiNet, and the analysis lacked adjustments for several potential confounders that were included in our study. The reason for teachers’ elevated risk is unclear, but previous research suggests that transmission in schools is likely and that teacher-to-teacher transmission is more common than student-to-teacher transmission.^27 28^ Transmission in schools may have contributed to our finding, given that there were several COVID-19 outbreaks in schools in the spring and autumn of 2020.^29^ In agreement with our finding, a previous study in Sweden^30^ reported that teachers who taught in-person at schools had two times higher risk of COVID-19 diagnosis than those who taught remotely.

Compared with Swedish-born workers in both non-essential and essential occupations, we found that foreign-born individuals regardless of occupational type had a higher risk of COVID-19 hospitalisation and ICU admission, even after controlling for several pre-existing commodities that are well-known determinants of severe COVID-19 outcomes. This finding aligns with previous studies^4 5 31^ and may reflect delayed healthcare-seeking behaviour or lack of access to timely healthcare due to cultural and economic reasons or language barriers.^32^

We found disproportionate risk of all three COVID-19 outcomes among foreign-born workers in essential occupations, which is in disagreement with a study from Norway^21^ that focused mainly on immigrants from five countries (Somalia, Pakistan, Iraq, Afghanistan and Turkey) and analysed only notified COVID-19 infections. To the best of our knowledge, our study is the first population-based study in Sweden to have examined the effects of immigration/occupation intersectional groups on all three COVID-19 outcomes (diagnosis, hospitalisation and ICU admission), and the results clearly suggest that occupation plays a substantial role in the burden of COVID-19 among foreign-born individuals in Sweden. A potential explanation for the finding may be the concentration of foreign-born individuals in less well paid essential occupations that are often associated with higher exposure to infections and less possibility of social distancing and access to adequate personal protective equipment.^13^ Because foreign-born individuals more commonly have temporary employments and lower income, the fear of losing their jobs could pressure them to continue working while unwell, thus increasing their risk of poor health outcomes.^33–35^ Frequent use of public transport could also contribute to the increased risk of COVID-19 outcomes among foreign-born workers in essential occupations who need to travel to be present at work.^33^

Our study has some limitations. First, individuals who were born to immigrant parents residing in Sweden were included in the category of Swedish-born population. This approach is likely to attenuate the associations, as this group of individuals might have lifestyle and living characteristics more similar to the foreign-born group, that may be associated with increased risk of COVID-19.^36^ Second, it was beyond the scope of our study to analyse the distribution of the risk across different immigrant groups. Hence, we cannot state whether the observed increased risk of COVID-19 outcomes among foreign-born workers in essential occupations, for example, is driven by an overrepresentation of foreign-born workers from a particular region. Third, although we controlled for a range of important potential confounders, we cannot rule out the possibility of residual confounding due to other unmeasured variables, such as work contracts and living conditions. Fourth, we defined our study sample based on an employment variable collected between 2018 and 2019. Some of the essential workers in our sample might have been included in the 1.6% of employed people that lost their jobs in 2020,^37^ which may lead to an underestimation of the true effect of occupation on COVID-19 outcomes. Fifth, we excluded participants with missing information on occupation. When we compared the characteristics of these participants with those who were included, our result revealed some differences mainly concerning sociodemographic and socioeconomic characteristics (online supplemental table A4). The observed differences might have introduced some selection bias although this is likely to be minimal and unlikely to greatly influence the study’s internal validity. Finally, our study sample was selected from a randomly sampled cohort of the Swedish population, with very slightly different age and gender structure in the studied age groups, which precludes an absolute direct transferability of our findings, but generalisability is still very good. Notwithstanding these limitations, our study has some notable strengths that include the prospective design, a large sample size and a relatively long follow-up. The use of register-based data minimises potential risk of non-differential misclassification and information bias. The study also has a wide coverage of COVID-19 cases that was made possible by the inclusion and use of COVID-19 data from different registers in Sweden.

10.1136/jech-2021-218278.supp4 Supplementary data

In summary, we found an increased risk of COVID-19 diagnosis, hospitalisation and ICU admission among essential workers. Healthcare workers had a higher risk of the outcomes than other essential workers. In comparing the risk of the three outcomes in foreign-born and Swedish-born workers in essential and non-essential occupations, we found that foreign-born workers in essential occupations had the most elevated risk of all the four groups compared, and that this risk was higher than what would be expected from independent risks. These findings were little influenced by additional adjustments for individual characteristics known to be risk factors for COVID-19, which suggests a need for further exploration of possible mechanistic factors underpinning the findings. The study findings support the ongoing government efforts to provide essential workers with resources to protect them against COVID-19. The study, however, underscores the importance of ensuring that COVID-19-related infection protection measures are tailored to meet the needs of foreign-born workers in essential occupations who tend to bear a double burden of risk because of their socioeconomic status.

**What is already known on this subject**

- Immigrants have disproportionate risk of COVID-19 and socioeconomic status has been suggested as potential explanatory factor.
- However, the contribution of occupation on the risk of COVID-19 diagnosis, hospitalisation and intensive care unit admission among immigrants in Sweden has not been studied.

**What this study adds**

- Working in essential occupations was associated with an increased risk of COVID-19 outcomes among employees in Sweden.
- When comparing the risk of COVID-19 diagnosis, hospitalisation and intensive care unit admission, in foreign- and Swedish-born workers in essential and non-essential occupations, we found that foreign-born workers in essential occupations had the most elevated risk of all the four groups compared.
- The findings support the ongoing government efforts to provide essential workers with resources to protect them against COVID-19 and underscore the importance of ensuring that COVID-19-related infection protection measures are tailored to meet the needs of foreign-born workers in essential occupations who tend to bear a double burden of risk because of their socioeconomic status.

## Data Availability

Data may be obtained from a third party and are not publicly available.
