# Supplementary material for: Occupation and COVID-19 diagnosis, hospitalisation and ICU admission among foreign-born and Swedish-born employees: a register-based study
Source: J Epidemiol Community Health. 2022 Jan 7;76(5):440–7. doi: 10.1136/jech-2021-218278 (PMC8761595; doi:10.1136/jech-2021-218278)
Supplement: Supplementary data [file jech-2021-218278supp001.pdf]

Online Supplementary Table A1: Essential occupations and codes based on the Swedish Standard for Classification of Occupations (SSYK 2012).

SSYK 2012	Occupations
Healthcare workers	
2211	Specialist physicians
2212	Resident physicians
2213	General medical practitioners
2219	Other physicians
2223	Anesthesia nurses
2224	District nurses
2226	Nurses- Ambulance
2227	Nurses- Geriatric
2228	Nurses- Intensive care
2231	Nurses- Operation
2232	Nurses- Pediatrics
2235	Nurses- Radiology
2239	Other specialist nurses
2260	Dentist
2271	Chiropractors and naprapaths
2272	Physiotherapists
2273	Occupational therapist
5321	Assistant nurses, home care and homes for the elderly
5322	Assistant nurse, rehabilitation
5323	Assistant nurses, hospital ward
5324	Assistant nurses, clinic
5325	Assistant nurses, pediatrics
5326	Ambulance attendants
5330	Home-based personal care and related workers
5350	Dental nurses
Education (teachers)	
2330	Secondary education teachers
2341	Primary school teachers
2342	Recreation leaders

2343	Preschool teachers
5311<br>5312	Childcare workers<br>Techers' aides
Service sectors	
5221<br>5222<br>5223<br>5224<br>5225<br>5226<br>5227	Shopkeepers and shop supervisors, selling in stores<br>Shop sales, groceries<br>Shop sales, specialty stores<br>Opticians' assistants<br>Petrol station personnel<br>Rental salespersons<br>Pharmacy technicians
5230	Cashiers and related clerks
7611<br>9411<br>9412<br>9413	Butchers, fishmongers and related food preparers<br>Pizza makers and fast-food preparers<br>Restaurant and kitchen helpers<br>Café and confectionery assistants
Police and security services	
3360	Police officers
5412	Prison guards
5413	Security guards
Postal workers and delivery	
4420	Postmen and postal facility workers
8329	Motorcycle, bicycle drivers and other automobile drivers not elsewhere classified
Transport services	
8321	Taxi, car, and van drivers
8331	Bus and tram drivers
Cleaners	
9111	Cleaners and helpers in offices, hotels, and other establishments
