# Supplementary material for: Occupation and COVID-19 diagnosis, hospitalisation and ICU admission among foreign-born and Swedish-born employees: a register-based study
Source: J Epidemiol Community Health. 2022 Jan 7;76(5):440–7. doi: 10.1136/jech-2021-218278 (PMC8761595; doi:10.1136/jech-2021-218278)
Supplement: Supplementary data [file jech-2021-218278supp002.pdf]

Online Supplementary Table A2. Pre-existing comorbidities and their International Classification of Disease codes

**Pre-existing comorbidities**	**ICD codes (version 10)**
Hypertension	110-115
Stroke	160, 161, 162, 1630-1635, 1638-1639, 164
Psychiatric conditions	F20 F21, F22, F23, F24, F25, F26, F27, F28, F29, F30, F31, F32, F33, F34, F35, F36, F37, F38, F39
Diabetes	E10, E11
Pneumonia	J10-J18
COPD	J44
Asthma	J45
Obesity	E66
