# Supplementary material for: Occupation and COVID-19 diagnosis, hospitalisation and ICU admission among foreign-born and Swedish-born employees: a register-based study
Source: J Epidemiol Community Health. 2022 Jan 7;76(5):440–7. doi: 10.1136/jech-2021-218278 (PMC8761595; doi:10.1136/jech-2021-218278)
Supplement: Supplementary data [file jech-2021-218278supp004.pdf]

Online Supplementary Table A4. Differences in study characteristics between selected and excluded participants aged 20-65 years and employed or self-employed

	Selected sample N = 326,052 %	Excluded sample N = 32,333 %	*P*-value
Age (years)			<0.001
20-34	28.33	38.18	
35-44	23.28	21.12	
45-54	24.31	20.20	
55-65	24.08	20.15	
Gender			<0.001
Men	49.73	60.52	
Women	50.27	39.48	
Healthcare region			<0.001
Stockholm	24.30	30.50	
Northern	8.83	8.01	
Southeastern	10.49	8.85	
Southern	17.21	17.23	
Uppsala-Orebro	20.06	18.51	
Western	19.11	16.91	
Immigrant status			<0.001
Swedish-born	81.84	72.21	
Foreign-born	18.16	27.79	
Marital status			<0.001
Married	43.14	37.73	
Single	45.05	50.85	
Divorced/widowed	11.81	11.42	
Highest education			<0.001
Primary	8.22	14.07	
Secondary	47.39	50.59	
Tertiary	44.39	35.33	
Individual annual gross income			<0.001
< 1000 SEK	6.54	30.37	
1000-2999 SEK	28.37	36.22	
3000-4999 SEK	46.64	24.83	
≥ 5000 SEK	18.45	8.59	
Hypertension			0.055
No	96.55	96.75	
Yes	3.45	3.35	
Diabetes			0.221
No	96.39	96.26	
Yes	3.61	3.74	
Obesity			0.853
No	98.25	98.23	
Yes	1.75	1.77	
Stroke			0.256
No	99.68	99.64	
Yes	0.32	0.36	
Pneumonia			0.478

No	98.97	98.93	
Yes	1.03	1.07	
COPD			0.547
No	99.81	99.80	
Yes	0.19	0.20	
Asthma			0.500
No	98.50	98.45	
Yes	1.50	1.55	
Psychiatric conditions			<0.001
No	97.01	95.72	
Yes	2.99	4.28	
