# Supplementary material for: Occupation and COVID-19 diagnosis, hospitalisation and ICU admission among foreign-born and Swedish-born employees: a register-based study
Source: J Epidemiol Community Health. 2022 Jan 7:jech-2021-218278. doi: 10.1136/jech-2021-218278 (PMC8761595; doi:10.1136/jech-2021-218278)
Supplement: Supplementary file 2 [file jech-2021-218278supp002.pdf]

Online Supplementary Table A2. Pre-existing comorbidities and their International Classification of Disease codes

| **Pre-existing comorbidities** | **ICD codes (version 10)** |
|---|---|
| Hypertension | 110-115 |
| Stroke | 160, 161, 162, 1630-1635, 1638-1639, 164 |
| Psychiatric conditions | F20 F21, F22, F23, F24, F25, F26, F27, F28, F29, F30, F31, F32, F33, F34, F35, F36, F37, F38, F39 |
| Diabetes | E10, E11 |
| Pneumonia | J10-J18 |
| COPD | J44 |
| Asthma | J45 |
| Obesity | E66 |
