# Supplementary material for: Occupation and COVID-19 diagnosis, hospitalisation and ICU admission among foreign-born and Swedish-born employees: a register-based study
Source: J Epidemiol Community Health. 2022 Jan 7:jech-2021-218278. doi: 10.1136/jech-2021-218278 (PMC8761595; doi:10.1136/jech-2021-218278)
Supplement: Supplementary file 3 [file jech-2021-218278supp003.pdf]

Online Supplementary Table A3: Unadjusted and adjusted associations between immigrant status and COVID-19 outcomes among individuals aged 20-65 years and employed or self-employed

	Unadjusted HR (95%CI)	*P*-value	Adjusted HR (95%CI)	*P*-value
**COVID-19 diagnosis**				
Swedish-born	1.00		1.00	
Foreign-born	1.33 (1.30-1.37)	<0.001	1.28 (1.24-1.32)	<0.001
**COVID-19 hospitalization**				
Swedish-born	1.00		1.00	
Foreign-born	3.09 (2.73-3.49)	<0.001	2.74 (2.40-3.13)	<0.001
**COVID-19 ICU admissions**				
Swedish-born	1.00		1.00	
Foreign-born	2.79 (2.01-3.87)	<0.001	2.53 (1.77-3.61)	<0.001

HR, hazard ratios; adjusted for age, gender, marital status, healthcare region, education, income, occupation, and pre-existing comorbidities (hypertension, diabetes, obesity, stroke, asthma, COPD, pneumonia, and psychiatric condition)
